# Waist Circumference and Its Association With Premenstrual Food Craving: The PHASE Longitudinal Study

**DOI:** 10.3389/fpsyt.2022.784316

**Published:** 2022-04-27

**Authors:** Nhan Dang, Dina Khalil, Jiehuan Sun, Aamina Naveed, Fatimata Soumare, Ajna Hamidovic

**Affiliations:** ^1^Department of Public Health, University of Illinois at Chicago, Chicago, IL, United States; ^2^Department of Public Health, Benedictine University, Lisle, IL, United States; ^3^Department of Pharmacy, University of Illinois, Chicago, IL, United States

**Keywords:** central obesity, food craving, premenstrual dysphoric disorder (PMDD), premenstrual syndrome (PMS), body mass index (BMI)

## Abstract

Visceral adiposity is a significant marker of all-cause mortality. Reproductive age women are at a considerable risk for developing visceral adiposity; however, the associated factors are poorly understood. The proposed study evaluated whether food craving experienced during the premenstrual period is associated with waist circumference. Forty-six women (mean BMI = 24.36) prospectively provided daily ratings of food craving across two-three menstrual cycles (122 cycles total). Their premenstrual rating of food craving was contrasted against food craving in the follicular phase to derive a corrected summary score of the premenstrual food craving increase. Study groups were divided into normal (*n* = 26) and obese (*n* = 20) based on the 80 cm waist circumference cutoff signifying an increase in risk. Waist circumference category was significantly associated with premenstrual food cravings [*F*_(1,44)_ = 5.12, *p* = 0.028]. *Post hoc* comparisons using the Tukey HSD test (95% family-wise confidence level) showed that the mean score for the food craving effect size was 0.35 higher for the abdominally obese *vs.* normal study groups (95% CI: 0.039 to 0.67). The result was statistically significant even following inclusion of BMI in the model, pointing to a particularly dangerous process of central fat accumulation. The present study establishes an association between temporal vulnerability to an increased food-related behavior and a marker of metabolic abnormality risk (i.e., waist circumference), thereby forming a basis for integrating the premenstruum as a viable intervention target for this at-risk sex and age group.

## Introduction

Obesity is considered a 21st century epidemic and reliance on the measurement of body mass index (BMI) alone has been shown to be inadequate in assessing and managing obesity-related cardiometabolic risk (1, 2). Waist circumference—a marker of visceral adiposity—is strongly associated with all-cause (3, 4) and cardiovascular (5, 6) mortality. Visceral fat accumulates when adipose tissue has a limited ability to expand. This process is indicative of a hyperlipolytic state; marked by resistance to insulin and an enhanced adipokine output.

There are large differences in body composition between men and women. With an overall weight gain, waist circumference also increases in young adults; however, men have larger increases in waist circumference naturally accompanying their overall greater weight gain compared to women. Hence, a universally acceptable approach is to set a higher circumference cutoff value for abdominal obesity in men compared to women (7). For example, the International Diabetes Federation sets the threshold for abdominal obesity for men at 90 cm (or 94 cm for White men), and 80 cm for women across ethnic backgrounds. Metabolic syndrome—providing clinicians with a simple screening tool to identify individuals who are likely to have insulin resistance and related metabolic abnormalities—lists waist circumference (≥ 80 cm in women) as one of the five possible criteria for the syndrome, highlighting the importance of central adiposity on disease probability (8, 9).

The premenstruum is marked by an increase in food craving in a significant number of reproductive age women, who are particularly vulnerable to weight gain (10–13). A study by Hartlage et al. (14) administered a questionnaire listing 50 premenstrual symptoms to women from the community and showed that food craving ranked second in magnitude, immediately after bloating. Indeed, food craving, defined as an “intense desire to eat a specific food or food type” (15, 16) is endorsed by women in the premenstruum both retrospectively (17, 18) and prospectively (14, 19, 20). Functional magnetic resonance tomography studies show higher brain responses to food stimuli in the luteal *vs.* follicular phase, particularly in the corticolimbic areas involved in homeostasis and reward (21–24). Combined, the results of this research characterize functional and self-reported indices of increased food craving in the premenstruum.

No study to date has established an association between either the PMS/PMDD diagnosis, or a particular premenstrual symptomatology, and central adiposity. Data from the Nurses’ Health Study 2 (NHS2) was analyzed for central adiposity (waist circumference); however, only an association between PMS and BMI was found. PMS cases were identified by asking women whether they ever received a diagnosis for PMS—an approach which may have diminished the power to isolate an association. PMS diagnosis is based on various symptom types (see Section “Diagnostic Criteria”), some of which may be distal to central adiposity. As only one symptom must be met (and up to the total of four), a woman would meet the PMS diagnosis if she reported, for example, increased difficulty concentrating in the premenstruum. An association with the PMS diagnosis in this case may be more difficult to capture than if the diagnosis was based on food craving. Moreover, PMS/PMDD diagnosis is rarely established clinically using prospective recordings across two menstrual cycles, as required in DSM-5; hence, it is possible that there were diagnostic misclassifications of the ever/never diagnostic status in the Bertone-Johnson et al. (25) analysis because the diagnosed participants were likely not followed prospectively at the time of the diagnosis determination.

Our analysis is based on a stringent prospective symptom data collection of premenstrual food craving symptomatology. Importantly, it is adjusted for BMI, which is necessary as the full strength of the association between waist circumference with morbidity and mortality is realized only after adjustment for BMI (3, 26, 27). Based on the literature showing that women’s food craving increases premenstrually (14), and that there is an association between food craving and waist circumference in women even independent of the reproductive status (28), we hypothesized that there would be a positive association between premenstrual food craving and waist circumference. Our study identified the premenstruum as a window of opportunity for the development of targeted interventions, which is of critical importance for health promotion in reproductive age women, who are especially vulnerable to developing obesity.

## Materials and Methods

### Study Design

Premenstrual Hormonal and Affective State Evaluation (PHASE) is a single-cohort longitudinal design study with a nested human laboratory experiment. The study enrolls women with regular menstrual cycles to chart their symptoms using the Daily Record of Severity of Problems (DRSP) (29), menstruation timing and urinary luteinizing hormone (LH) levels during three menstrual cycles. In the third menstrual cycle, in addition to DRSP collection and LH testing, study participants complete: (1) blood and salivary sample collection at eight different times of the menstrual cycle (early follicular, mid-follicular, periovulatory 1, periovulatory 2, periovulatory 3, early luteal, mid-luteal, and late luteal), and (2) psychosocial stress testing in the late luteal phase. Knowledge gained from PHASE is expected to increase our understanding of PMS/PMDD etiology. PHASE is a registered clinicaltrials.gov study (NCT03862469).

### Study Sample

Women between the ages of 18 and 35, with regular menstrual cycles lasting 21 to 35 days (30–33), were recruited from the general population using electronic media (Instagram, and Facebook) and flyers. The advertisement specified that the objective of the study is to evaluate “what causes” PMS.

Study participants first completed an online survey, following which they were scheduled to complete an in-person screening. Study exclusion criteria were: (a) lifetime DSM-5 Axis I disorder, except anxiety and depression [based on the Structured Clinical Interview for DSM Disorders (SCID)], (b) current (i.e., within the past 12 months) DSM-5 Major Depressive Disorder or an anxiety disorder (based on SCID), (c) positive urine drug screen test, (d) breath alcohol concentration > 0.00%, (e) Alcohol Use Disorders Identification Test (AUDIT) score > 7, (e) self-reported smoker or carbon monoxide concentration ≥ 6 ppm, (f) irregular menstrual cycle, (g) current pregnancy (urine test-verified) or lactation, or a plan to become pregnant, (h) moderate or high suicide risk, (i) Shipley IQ (vocabulary standard score) < 80, (j) prescription medications, and (k) hormonal contraception.

At the screening visit, study participants’ anthropomorphic measures were taken. Once enrolled and begun their subsequent menstrual cycle, study participants completed DRSP, as described in the next section.

### Study Measures

Food Cravings. The Daily Record of Severity of Problems (DRSP) (29) is a validated questionnaire which measures 24 symptoms of PMS/PMDD (affective, psychological, behavioral, and functional). The symptoms are rated on a scale of 1 (not at all) to 6 (extreme). The present analysis evaluated ratings of food craving (“Had cravings for specific foods”). Once enrolled, study participants notified the research coordinator when their next menstrual cycle started. Upon notification, the research coordinator set up the DRSP surveys to be sent out daily for the total of 2 menstrual cycles (3 menstrual cycles if the participant chose to complete the third menstrual cycle procedures). Study participants received a new survey link every day and were asked to complete it between 7 PM and midnight. They were also asked to use the day’s link and not any previous links to minimize retrospective reporting. In case a participant missed more than two DRSP entries in a row, or four or more in a month, the research coordinator contacted the participant to complete the survey daily.

Anthropomorphic Measures. Participants removed their shoes, hats, or any head gear when their height was measured using a standard measuring board with a moveable stadiometer. They removed outer layer of clothing and shoes prior to stepping on the digital scale for weight measurement. When measuring waist circumferences, participants were asked to cross their arms and place them on opposite shoulders. Waist circumference was measured by palpating the hip area to locate the right ilium of pelvis and positioning the tape in a horizontal place at the level of the measurement mark. The measurements were made with the tape held snugly, but not constricting, and at a level parallel to the floor.

### Diagnostic Criteria

The Diagnostic and Statistical Manual of Mental Disorders, Fifth Edition (DSM-5) lists food craving as one of the symptoms (out of the total 11) for premenstrual dysphoric disorder (PMDD) and premenstrual syndrome (PMS) diagnoses. The 11 symptoms may be affective, cognitive, behavioral, or physical in nature. Premenstrual Dysphoric Disorder (PMDD) requires presence of at least one affective symptom to reach the total of five required symptoms, which must be present in most cycles from the past year and confirmed in a prospective manner for at least two menstrual cycles. In addition, the symptoms have to be associated with clinically significant distress or interference with work, school, usual social activities, or relationship with others. Premenstrual syndrome requires presence of 1–4 symptoms, without the requirement that one must be affective in nature.

### Statistical Analysis

All analyses were performed in R software (version 4.0.2) (34). To compute premenstrual increase in food cravings, we averaged food craving ratings from the fifth to the tenth day from the start of the menstrual cycle and subtracted it from the average food craving ratings 5 days prior to thru and including the first day of the menstrual cycle. This value was then divided by a participant-specific variance across the entire menstrual cycle, yielding the participant-specific effect size (14). An effect size greater than 1.0 reflects presence of a particular PMS symptom (14).

We first assessed premenstrual food craving score for normality using the Shapiro–Wilk normality test. We categorized waist circumference as “normal” vs. “obese,” based on the 80-centimeter cutoff according to the International Diabetes Federation consensus guideline (35). BMI was analyzed as a categorical variable (≤ 24.9 “underweight/normal” and (≥ 24.9 “overweight/obese”). Race was analyzed as “White” *vs.* non-White.

The groups were compared on demographic characteristics using chi square tests for categorical, and independent 2-group *t*-test for continuous variables. We then assessed whether the waist circumference groups differed with respect to premenstrual food cravings. For this, we used the Analysis of Variance, with premenstrual food craving as the outcome and waist circumference (normal vs. obese) as the predictor followed by Tukey Honestly Significant Differences for the *post hoc* comparisons. The model was checked for homogeneity of variance (using the Levene Test) and normality of residuals’ distribution (using the Shapiro Test). In the next step (Analysis of Covariance), additional variables were added: BMI and race (White vs. non-White). The significance value was set at ≤ 0.05. For descriptive purposes, we also reported percent increases in premenstrual food cravings for abdominally obese vs. normal study groups. Additionally, for interpretability, we report analysis of variance and analysis of covariance results on premenstrual increase (5 days prior to thru and including the first day of the menstrual cycle) relative to the fifth to the tenth day from the start of the menstrual cycle, without adjustment for variance across the menstrual cycle.

## Results

### Study Participants

Forty-six participants, approximately 26 years old on average, contributed data for the present study. Based on the prospective rating of symptoms using the DRSP, twenty-six participants were in the normal group and twenty participants were in the obese group. Seven study participants met the diagnosis for PMDD, 19 for PMS, and 20 were healthy. The effect size and standard deviation for food craving ratings were 0.63 and 0.55. The participant sample was mostly White and Asian of non-Hispanic ethnicity. Approximately half of the participants were students. The average age of menarche was 12 years old. Table 1 lists demographic and anthropomorphic characteristics of study participants according to the group category. Study groups did not differ on any demographic characteristics or age of menarche.

**TABLE 1:** Demographic and anthropomorphic characteristics of study groups.

Variable	Group		*p* value
	
	Normal (*n* = 26)	Obese (*n* = 20)	
Age	25.73 (5.14)	25.95 (4.70)	0.882
Race			0.106
White	7 (26.9)	10 (50.0)	
African American	5 (19.2)	2 (10.0)	
American Indian/Alaska Native	0 (0.0)	1 (5.0)	
Asian	12 (46.2)	5 (25.0)	
More than 1 race	0 (0.0)	2 (10.0)	
Unknown	2 (7.7)	0 (0.0)	
**ETHNICITY**			
Hispanic	2 (7.7)	5 (25.0)	0.143
Non-hispanic	22 (84.6)	15 (75.0)	
Unknown	2 (7.7)	0 (0.0)	
Student status			
No	11 (42.3)	9 (45.0)	1.000
Yes	15 (57.7)	11 (55.0)	
**Marital status**			
Single/Never married	24 (92.3)	18 (90.0)	1.000
Married	2 (7.7)	2 (10.0)	
**Income**			
< $20,000	14 (53.8)	10 (50.0)	0.339
$20,000–$34,999	5 (19.2)	2 (10.0)	
$35,000–$49,999	3 (11.5)	3 (15.0)	
$50,000–$74,999	2 (7.7)	5 (25.0)	
$75,000 or more	2 (7.7)	0 (0.0)	
**Insurance**			
Government funding	4 (15.4)	4 (20.0)	0.519
Private insurance	18 (69.2)	15 (75.0)	
Self-pay or out of pocket	4 (15.4)	1 (5.0)	
Menarche age	12.00 (1.20)	12.13 (0.92)	0.725
Height (cm)	161.89 (7.99)	163.59 (6.52)	0.442
Weight (kg)	55.88 (6.45)	74.78 (11.85)	< 0.001
BMI	21.32 (2.00)	27.90 (3.87)	< 0.001
Sitting knee (cm)	47.68 (3.38)	49.05 (2.47)	0.134
Waist circumference (cm)	71.61 (4.59)	90.06 (11.09)	< 0.001
Arm circumference (cm)	26.15 (2.07)	31.96 (3.41)	< 0.001
Thigh circumference (cm)	48.45 (3.46)	55.85 (5.76)	< 0.001

*Values for continuous variables are expressed as mean (SD). Categorical variable values are expressed as N (%).*

### Association Between Food Craving and Waist Circumference

**FIGURE 1:**
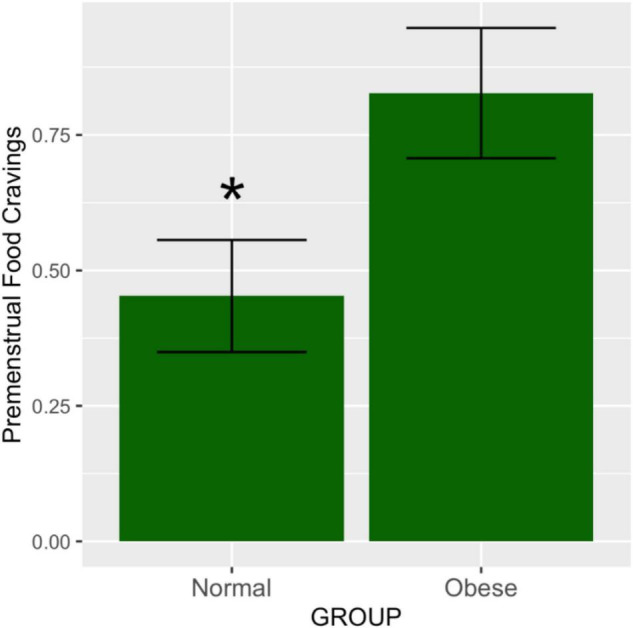
Premenstrual food cravings according to abdominal obesity groups. The means and SEM represent food cravings scores in the premenstrual vs. the mid-follicular subphase, adjusted for total variance across the entire menstrual cycle. As demonstrated by the ANOVA test, the obese group had significantly higher ratings of food cravings in the premenstruum compared to the non-obese group (*p* = 0.02). *p ≤ 0.05.

The effect size means and standard deviations for food cravings were 0.47 (0.51) and 0.82 (0.53) for the abdominally normal and obese groups, respectively. Waist circumference category was significantly associated with premenstrual food cravings [*F*_(1,44)_ = 5.12, *p* = 0.028]. *Post hoc* comparisons using the Tukey HSD test (95% family-wise confidence level) indicated that the mean score for the food craving effect size was 0.35 higher for the abdominally obese vs. normal study groups (95% CI: 0.039 to 0.67, p-adjusted = 0.028) (Figure 1). Table 2 lists the ANOVA model details. The Levene’s Test for Homogeneity of Variance (center = median) showed that the study groups had similar variance [*F*_(1,43)_ = 0.07, *p* = 0.79]. The Shapiro–Wilk normality test showed that residuals had normal distribution (*W* = 0.99, *p* = 0.96).

**TABLE 2:** Result summary of the ANOVA model for premenstrual food cravings.

	Degrees of Freedom	Sum Sq	Mean Sq	*F* value	Pr(>*F*)
Waist Category	1	1.42	1.41	5.12	0.0285*
Residuals	44	12.18	0.2768		

*p ≤ 0.05.

In the ANCOVA model, waist circumference category was significantly associated with premenstrual food cravings [*F*_(1,41)_ = 5.10, *p* = 0.029]. *Post hoc* comparisons using the Tukey HSD test (95% family-wise confidence level) indicated that the mean score for the food craving effect size was 0.35 higher for the abdominally obese vs. normal study groups (95% CI: 0.05 to 0.65; p-adjusted = 0.021). Participants’ BMI was also associated with premenstrual food cravings [*F*_(1,41)_ = 6.87, *p* = 0.012], however, this significance did not pass the adjustment (p-adjusted = 0.085). Table 3 lists the ANCOVA model details. The Shapiro–Wilk normality test showed that residuals had normal distribution (*W* = 0.99, *p* = 0.98).

**TABLE 3:** Result summary of the ANCOVA model for premenstrual food cravings.

	Degrees of Freedom	Sum Sq	Mean Sq	*F* value	Pr(>*F*)
Waist category	1	1.420	1.4198	5.699	0.0216*
BMI category	1	1.712	1.7123	6.873	0.0121*
Race	1	0.002	0.002	0.009	0.9232
Residuals	42	10.464	0.2491		

*p ≤ 0.05.

In relative terms, the mean and standard deviation percent change in premenstrual food cravings was 37.33 (41.43) for the normal group and 80.16 (84.47) for the abdominally obese group. Table 4 shows results of analysis of variance (and analysis of covariance) evaluating mean scores for the two groups.

**TABLE 4:** Mean Effect Size and Percent Change of Premenstrual Food Craving Increase for the Normal and Obese Study Groups (Unadjusted and Adjusted Models).

Model	Variable	Level	Mean effect size	Mean percent change	*p*-value
Unadjusted	Waist circumference category	Obese	0.827	80.163	0.028
		Normal	0.473	37.333	
Adjusted	Waist circumference category	Obese	0.827	80.163	0.021
		Normal	0.473	37.333	
	Race	White	0.682	64.306	0.9981
		Non-White	0.595	51.059	
	BMI category	Overweight/Obese	0.628	53.567	0.012
		Underweight/Normal	0.627	57.228	

## Discussion

In accordance with our hypothesis, the present study shows a positive association between premenstrual food craving and waist circumference. This association remained significant even after adjusting for BMI, suggesting a particularly dangerous central fat accumulation. Though preliminary in nature, this is the first study to link food craving in the premenstruum with central adiposity.

Changing sex hormones in the luteal phase are associated with decreased levels of amino acids and lipid species, presenting a physiological milieu of heightened energy requirement (36–41) and indicative of an anabolic state (41, 42). The amino acid decrease likely reflects the effect of rising progesterone levels in the luteal phase. Progesterone upregulates cell cycle and growth, resulting in protein biosynthesis for endometrial thickening (43). Therefore, women may feel an increase in food craving during this time to satisfy this underlying energy expenditure. In one study (41), as many as 39 amino acids were suppressed in the luteal phase. A potential relationship between premenstrual amino acid levels, food craving, eating patterns and central adiposity should be investigated in future studies. Understanding which amino acid suppression is potentially linked to heightened premenstrual food craving may provide a basis for a targeted intervention development.

Alternatively, the relationship observed in the present study is driven by increased food intake as a means of affect regulation in the premenstruum, though this hypothesis should be tested. Reward-driven behaviors are goal-oriented changes and are based on past experiences as well as positive associations we may make with food (44, 45). If eating provides a euphoric experience, the behavior to consume this food will be reinforced and repeated. Therefore, craving, operationalized as appetitive motivation, can be portrayed as the amount of work and effort an individual is willing to perform to obtain a type of food. Our finding possibly reflects deficiency in reward-driven behaviors in a segment of women with PMS/PMDD, who score high on reward dependence (46), show low positive affect (47, 48) as well as low frontal electroencephalography (49–51), which are associated with deficient reward processing (52, 53).

Temporal behavioral interventions should be developed in the event subsequent studies demonstrate that the association observed in the present study is mediated *via* an increase in food intake. Reducing central adiposity in reproductive age women would have a population-level impact, as interventions would improve not only their health and well-being, but also that of their offspring (54). However, unless carefully designed, such efforts may fail. Generic interventions are generally not successful in reproductive age women, as evidenced by lower recruitment and retention rates, inferior attendance and compliance, and worse weight loss outcomes in comparison to older adult participants (55).

There is some, though limited, evidence that individualized treatment in reproductive age women is effective. For example, Eiben et al. (56) targeted diet, exercise, and behavior change utilizing face-to-face contact followed by ongoing regular telephone and e-mail contact for 1 year (56), and showed reduced central adiposity. Levitskey and colleagues (57) showed that a minimal intervention, where young women weighed themselves daily for 10 weeks and were provided with feedback on energy needs for weight maintenance, resulted in weight stability compared with controls who gained weight. However, long-term evaluation of these or other behavioral interventions in reproductive age women is not known (58) and no studies to date incorporated premenstrual behavioral changes related to food cravings. Results of our study provide compelling evidence that an intervention designed to specifically target premenstrual cravings may result in an efficacious reduction of central adiposity given that food cravings may be conditioned by interventions (59–64).

By calculating effect size for premenstrual food craving, our goal was to emphasize its importance as a clinical problem due to its link to central adiposity. We show that nutrient craving is affected by changing sex hormones between menstrual phases, which may predispose a segment of women to high nutritional needs. The mean effect size for premenstrual food craving in our study was 0.69, while in the study by Hartlage et al. (14), it was 0.29 for the community sample and 0.96 for the PMDD clinical sample. The effect size difference in our study is likely due to our advertising strategy, which asked “Do you have PMS?,” drawing a sample of participants with PMS/PMDD symptomatology from the community pool.

The present study is the first to identify a segment of reproductive age women who have an adverse health marker (waist circumference) related to premenstrual food craving. The findings of our study are preliminary in nature, and future evaluations should be completed in larger participant samples. If replicated, the finding of the present study provides a platform for further novel nutrition interventions, with an emphasis on improved health and well-being in this at-risk population.

## Data Availability Statement

The original contributions presented in the study are included in the article/supplementary material. Further inquiries can be directed to the corresponding author.

## Ethics Statement

This study protocol was reviewed and approved by the Office for the Protection of Research Subjects (OPRS) at the University of Illinois at Chicago, approval number 2018-1533. The patients/participants provided their written informed consent to participate in this study.

## Author Contributions

ND analyzed the study data. DK, AN, and FS assisted with the manuscript preparation and submission. JS provided the statistical consulting. AH designed the study, analyzed the study data, and wrote the initial and final manuscript versions. All authors contributed to the article and approved the submitted version.

## Conflict of Interest

The authors declare that the research was conducted in the absence of any commercial or financial relationships that could be construed as a potential conflict of interest.

## Publisher’s Note

All claims expressed in this article are solely those of the authors and do not necessarily represent those of their affiliated organizations, or those of the publisher, the editors and the reviewers. Any product that may be evaluated in this article, or claim that may be made by its manufacturer, is not guaranteed or endorsed by the publisher.
